# A Hop Extract Lifenol® Improves Postmenopausal Overweight, Osteoporosis, and Hot Flash in Ovariectomized Rats

**DOI:** 10.1155/2018/2929107

**Published:** 2018-02-12

**Authors:** Young-Hwan Ban, Jung-Min Yon, Yeseul Cha, Jieun Choi, Eun Suk An, Haiyu Guo, Da Woom Seo, Tae-Su Kim, Sung-Pyo Lee, Jong-Choon Kim, Ehn-Kyoung Choi, Yun-Bae Kim

**Affiliations:** ^1^College of Veterinary Medicine and Veterinary Medical Center, Chungbuk National University, Cheongju, Republic of Korea; ^2^Anydoctor Healthcare Co., Ltd., Cheonan, Republic of Korea; ^3^College of Veterinary Medicine, Chonnam National University, Gwangju, Republic of Korea

## Abstract

**Objective:**

In order to assess the effectiveness of a hop extract (HE) for postmenopausal symptoms, the effects of Lifenol on ovariectomy-induced osteoporosis, hyperlipidemia, body weight increase, and hot flash were investigated in rats.

**Methods:**

Female Sprague-Dawley rats were ovariectomized and subjected to a daily scheduled exercise training (15 min at 15 m/min) or treated with HE (30 or 100 mg/kg, oral) or 17*β*-estradiol (100 *μ*g/kg, intraperitoneal) for 12 weeks. Body and visceral fat weights, serum lipid profiles, osteoporotic parameters in serum, and femoral bones were analyzed. Separately, forced running-induced dermal and rectal temperatures and blood flow velocity were measured in ovariectomized rats.

**Results:**

Ovariectomy increased blood lipids including triglycerides, total cholesterol, and low-density lipoproteins, leading to visceral fat accumulation and overweight. Estrogen depletion caused osteoporosis, displaying decreased femoral bone weight, bone mineral density and content, and blood phosphorus level. The disturbances in lipid metabolism and bone resorption were recovered by treatment with HE in a dose-dependent manner. In addition, HE treatment shortened the duration of forced running-induced alterations in skin and rectal temperatures by reducing blood flow velocity.

**Conclusion:**

The results indicate that HE attenuated overweight, osteoporosis, and hot flash in estrogen-deficient animals by regulating blood lipid profile and fat accumulation, blood estrogen and bone resorption factors, and dermal blood flow.

## 1. Introduction

Menopausal women commonly experience symptoms including osteoporosis, hot flash, hyperlipidemia, cardiovascular disease, and fat redistribution, which are associated with decrease in endogenous estrogen level [1, 2]. Estrogen deficiency facilitates osteoporosis by lowering bone mass of trabecular bones, leading to increased fragility, which is due to the imbalance between bone resorption and bone formation [3]. At menopause, there are increases in abdominal fat and cardiovascular disease risk factors associated with a worsening of blood lipid profile, that is, high levels of triglycerides (TG), total cholesterol (TC), and low-density lipoproteins (LDL) [4–6]. Hot flash, related to change in blood circulation, is a quick feeling of heat and sometimes a red, flushed face and sweating due to the disturbances in hypothalamic control of temperature [7]. The characteristic of hot flash is dermal vasodilatation, leading to increased skin blood flow (flushing) and skin temperature, although sometimes there may be a transient decrease in core temperature [8].

Hormone replacement therapy (HRT), composed of estrogen and progesterone, has been used to treat the climacteric complaints [9], but HRT is commonly associated with side effects such as breast cancer and heart disease as long-term effects [10]. Hence, natural selective estrogen receptor modulators have been recommended to reduce those discomforts. The effects of plant-based remedies, such as soy, red clover, flaxseed, black cohosh, and chaste tree berry, are from their constituents known as phytoestrogens. Phytoestrogens are polyphenolic nonsteroidal plant compounds with estrogen-like biological activity. They exert such activity by directly binding to estrogen receptors or by influencing the production, metabolism, and action of natural hormones at the cellular level [11, 12].

Hop (*Humulus lupulus *L.) in Cannabaceae family has been used as a bitter constituent of beer worldwide. Notably, hop extracts contain diverse phytoestrogen compounds that were the highest in the extracts from female flowers of the hop plant. As active components (phytoestrogens) of hop plant, prenylflavonoids including 8-prenylnaringenin (8-PN), isoxanthohumol (IX), and xanthohumol have been demonstrated, in which 8-PN was the most potent ingredient with binding affinity to estrogen receptors [13]. The estrogenic activity of 8-PN was found to be higher than that of well-established phytoestrogens such as coumestrol, genistein, and daidzein [14].

In the present study, we investigated the effectiveness of a hop extract (HE) in the improvement of postmenopausal symptoms including osteoporosis, hyperlipidemia, body weight increase, and hot flash in ovariectomized (OVX) rats.

## 2. Materials and Methods

### 2.1. Materials

The dried HE was provided by Anydoctor Healthcare Co., Ltd. (Cheonan, Chungnam, Korea), which was originally from Lifenol extract (Naturex, Avignon, France). For the preparation of Lifenol, dry hop cones (*Humulus lupulus* L.) were extracted for 2 hours under supercritical CO_2_ conditions (60°C, pressure 400 bar) [15, 16] followed by 75% ethanol extraction, concentrated with a vacuum evaporator, and spray-dried after adding silica E 551 (final yield = 6%). In HPLC analysis, the concentration of 8-PN as a functional ingredient in Lifenol was 0.1–0.2% that is much higher than the concentration (approximately 0.01%) in original hop cones.

### 2.2. Animals

Eleven-week-old female Sprague-Dawley rats (body weights 240–250 g) were purchased from Daehan Biolink (Eumseong, Chungbuk, Korea). They were housed in a room with constant temperature (23 ± 3°C), relative humidity (50 ± 10%), and 12-hour light cycle. Animals were fed a standard commercial rodent chow (Daehan Biolink) and purified water* ad libitum*. All animal experiments were in accordance with the Standard Operation Procedures of Laboratory Animal Research Center, Chungbuk National University (CBNU), Korea, and the protocols were approved by the Institutional Animal Care and Use Committee of CBNU (approval number CBNUA-630-13-01).

### 2.3. Ovariectomy and Treatment

The rats except Sham-operation group were ovariectomized at the age of 3 months. Three weeks after recovering from the surgery, the OVX rats were randomly divided into five groups (*n* = 7/group): OVX alone, scheduled exercise training (Ex), low dose (30 mg/kg) HE (HE30), high dose (100 mg/kg) HE (HE100), and 17*β*-estradiol (E2, 100 *μ*g/kg) treatment groups. Ex group animals were subjected to a scheduled exercise on a motor-driven treadmill (MK-680S; Muromachi Kikai, Tokyo, Japan) for 15 min at 15 m/min every day for 12 weeks. HE30 and HE100 group rats were orally administered once a day with 30 and 100 mg/kg HE, respectively, and E2 group animals were given 17*β*-estradiol valerate (Sigma-Aldrich, St. Louis, MO, USA) intraperitoneally, for 12 weeks.

Weekly body weight was recorded. After 12-week treatment, the rats were sacrificed. Arterial blood was collected, and serum was stored at −20°C for analysis of hormone and biochemical parameters. Collected left femur was stored at 4°C for analysis of bone mineral density (BMD) and bone mineral content (BMC), and right femur was fixed in 10% formalin solution for haematoxylin-eosin staining and tartrate-resistant acid phosphatase (TRAP) immunostaining. Visceral (perirenal, retroperitoneal, and mesenteric) fats were collected and weighed.

### 2.4. Blood Hormone and Biochemical Analysis

Serum 17*β*-estradiol concentration was analyzed using Coat-A-Count® Estradiol Kit (Diagnostic Products Co., Los Angeles, CA, USA). Biochemical analysis was performed using an automatic analyzer (INTEGRA 400; Roche, Mannheim, Germany) for calcium (Ca), inorganic phosphorus (P), and alkaline phosphatase (ALP) as well as lipids including TG, TC, LDL, and high-density lipoproteins (HDL).

### 2.5. Determination of Femoral BMD and BMC

Left femur of the rats was excised, cleaned of adhering soft tissues, and preserved in 10% formalin solution. BMD and BMC were assessed via a dual-energy X-ray absorptiometry (DEXA) specifically designed for small animals (PIXImus II Densitometer; LUNAR, Madison, WI, USA).

### 2.6. Microscopic Examination

Right femurs were decalcified in 10% EDTA at 4°C for 14 days. Paraffin-embedded sections (5 *μ*m in thickness) were prepared and stained with hematoxylin-eosin. For TRAP immunostaining, a part of the sections was reactivated in 0.2 M Tris-HCl buffer and incubated with TRAP medium for 1.5 hours at room temperature [17]. The sections were counterstained with 1% methyl green. The areas of growth plate and TRAP-positive tissues were analyzed with Image J software (version 1.50a; National Institutes of Health, Bethesda, MD, USA).

### 2.7. Measurement of Dermal Temperature, Rectal Temperature, and Blood Flow Velocity

Another set of OVX rats was pretreated with HE (30 or 100 mg/kg) or E2 (100 *μ*g/kg) for 14 days. Thirty min after final administration, the rats were subjected to a forced running on a motor-driven treadmill (MK-680S; Muromachi Kikai, Tokyo, Japan) for 15 min at a speed of 15 m/min. Immediately after the forced running, the tail skin and rectal temperatures were measured for 120 min at 10-min intervals, and the net changes were calculated by subtracting original (mean temperature during 20 min before forced running) from those of each time point. Tail skin temperature was measured with the Tail Flick Test Analgesia Meter (33T; IITC Life Science, Woodland Hills, CA, USA) on the dorsal surface of the tail, approximately 1 cm from the base [18]. Rectal temperature was measured with TH-5 Thermalert Monitoring Thermometer (Physitemp Instruments, Clifton, NJ, USA). Immediately after the 6th measurement of dermal temperature (60 min after forced running), blood flow velocity in the tail was measured at 8 cm from the base using a laser tissue blood flowmeter (FLO-C1; Omegawave, Espoo, Finland).

### 2.8. Statistical Analysis

Data were expressed as mean ± SEM. Statistical significance among test groups was determined by one-way analysis of variance followed by the Tukey's and Duncan's tests using the SPSS 10.0 statistics computer program (SPSS, Chicago, IL, USA). A difference in the mean values of *P* < 0.05 was considered to be statistically significant.

## 3. Results

### 3.1. Effects on Body Weight, Fat Weight, and Serum Lipid Profile of OVX Rats

**Figure 1:**
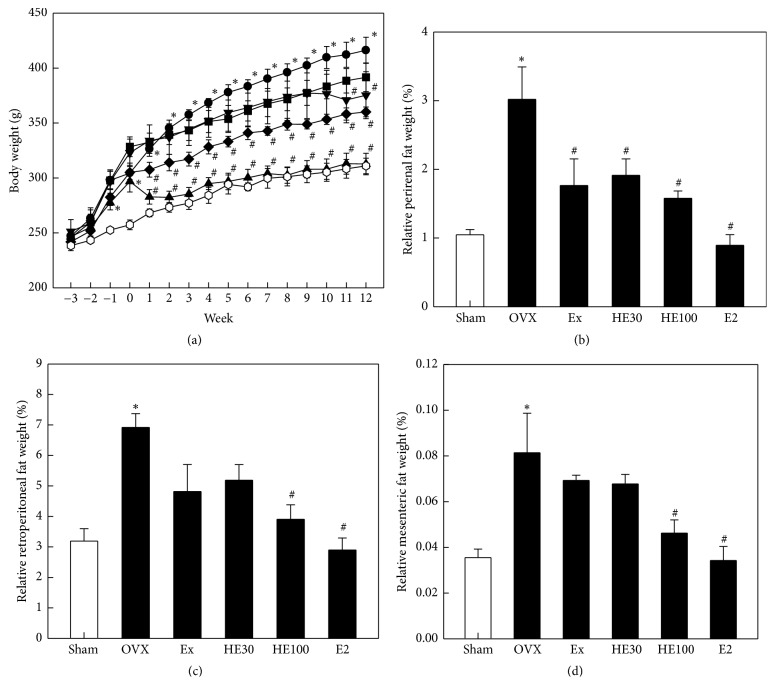
Effects of scheduled exercise training (Ex), hop extract (HE), and 17*β*-estradiol (E2) on the body weight change and visceral fat weights in ovariectomized (OVX) rats. ○: Sham-operation control, ●: OVX alone; ▼: Ex, ■: HE30 (30 mg/kg), ◆: HE100 (100 mg/kg), and ▲: E2 (100 *μ*g/kg). ^*∗*^Significantly different from Sham-operation control (*P* < 0.05). ^#^Significantly different from OVX alone (*P* < 0.05).

During 3-week recovery time after OVX, the body weight gain in OVX rats was significantly higher than that in Sham-operated animals (*P* < 0.05) (Figure 1(a)). Such an increasing pattern of body weight change was continued during the 12-week experimental period. The OVX-induced body weight gain was attenuated by scheduled exercise or oral administration of a low dose of HE (HE30) to some extent. Notably, a high dose of HE (HE100) significantly suppressed the body weight gain from 1 week of treatment (*P* < 0.05). By comparison, injection of E2 significantly decreased the body weight for 2 days (*P* < 0.05), and thereafter leading to the pattern of Sham-operation group.

The weights of perirenal, retroperitoneal, and mesenteric fats in OVX rats markedly increased to 2.2–2.9-folds control levels in Sham-operated animals (*P* < 0.05) (Figures 1(b)–1(d)). Scheduled exercise reduced the OVX-induced increase in fat weight, in which the highest effect was observed in perirenal fats. It is of interest to note that HE decreased the fat weight in a dose-dependent manner. In comparison, E2 administration fully suppressed the fat weight to the control levels.

Ovariectomy significantly increased the blood TG, TC, and LDL levels, enhancing LDL/HDL ratio (*P* < 0.05) (Table 1). Scheduled exercise fully recovered TG concentration to the control level, although other lipids were reduced to some extent. Notably, HE remarkably reduced all the 3 lipids, without affecting the HDL level, leading to a decreased LDL/HDL ratio. By comparison, E2 greatly decreased TC and LDL levels (*P* < 0.05), but not TG level. Although E2 decreased LDL and LDL/HDL ratio, it also seriously lowered HDL level.

### 3.2. Effects on Osteoporosis and Related Parameters

**Figure 2:**
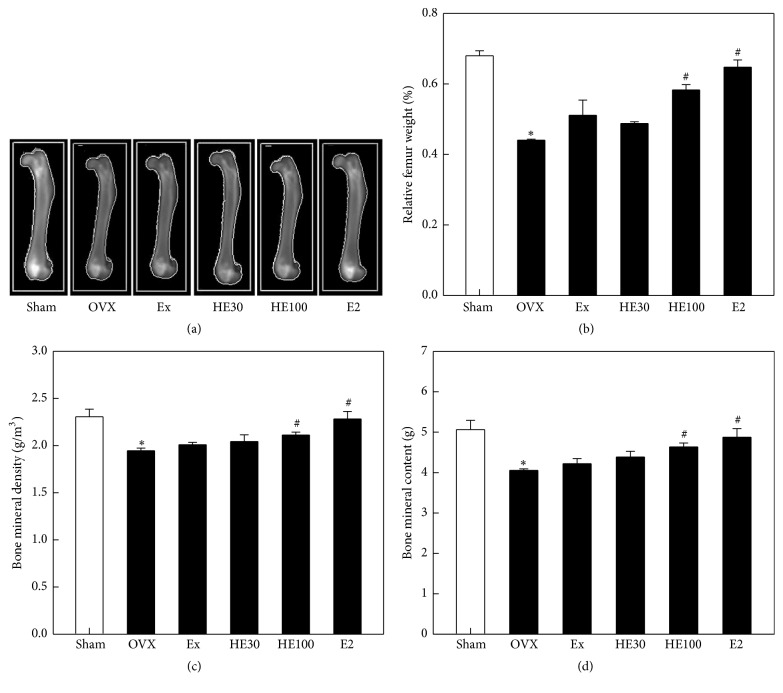
Effects of scheduled exercise training (Ex), hop extract (HE), and 17*β*-estradiol (E2) on the femur weight and bone mineral density and content analyzed by dual-energy X-ray absorptiometry (a) in ovariectomized (OVX) rats. ^*∗*^Significantly different from Sham-operation control (*P* < 0.05). ^#^Significantly different from OVX alone (*P* < 0.05).

In dual-energy X-ray absorptiometry, the femurs showed different densities according to OVX and/or treatment with scheduled exercise, HE, and E2 (Figure 2(a)), in parallel with their weights (Figure 2(b)). The relative femur weight (% of body weight) decreased by 35% following OVX, resulting from the significant decreases in BMD and BMC (*P* < 0.05) (Figures 2(c) and 2(d)). However, the decreased femur weight, BMD, and BMC were significantly recovered by treatment with a high dose of HE100 and E2 (*P* < 0.05).

After ovariectomy, the 17*β*-estradiol level greatly decreased (Table 2). Among the parameters affecting the bone metabolism, ALP (a key enzyme for bone turnover) significantly increased, while P (a bone-strengthening mineral) decreased (*P* < 0.05), although Ca was not altered. Scheduled exercise increased blood 17*β*-estradiol level and decreased ALP activity, although there were no significant changes. Notably, HE markedly recovered 17*β*-estradiol concentration and suppressed ALP activity (*P* < 0.05). E2 also restored 17*β*-estradiol level with a mild inhibition of ALP activity. All the treatments did not affect the blood Ca and P levels.

**Figure 3:**
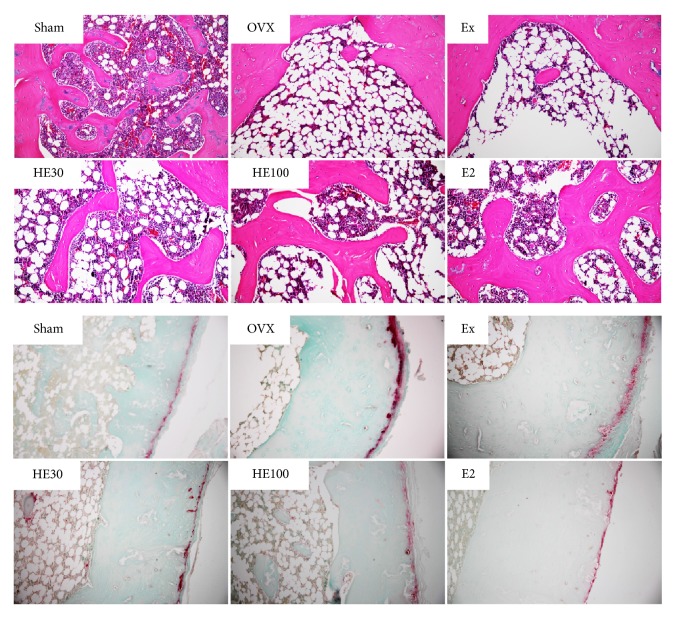
Representative microscopic findings (upper panel) and tartrate-resistant acid phosphatase (TRAP) immunoreactivity in the distal femoral bones of ovariectomized (OVX) rats. Note the trabecular bone loss and strong TRAP (an osteoclastic marker) immunoreactivity in OVX animals.

In the microscopic examination on the hematoxylin-eosin-stained distal femoral bones, well-formed bone masses with well-connected trabeculae were observed in the Sham-operated rats (Figure 3, upper panel). In the OVX rats, however, severe losses of the trabecular bones were observed. In comparison with the negligible effect of scheduled exercise, treatment with HE or E2 exhibited remarkable restoration of the trabecular structure. In the immunostaining of TRAP, an osteoclast-specific enzyme, a strong immunoreactivity was revealed in OVX rats compared with that in Sham group animals (Figure 3, lower panel). Scheduled exercise exhibited a week reducing activity. By comparison, HE and E2 strongly inhibited the osteoclast activation.

### 3.3. Effects on Hot Flash of OVX Rats

**Figure 4:**
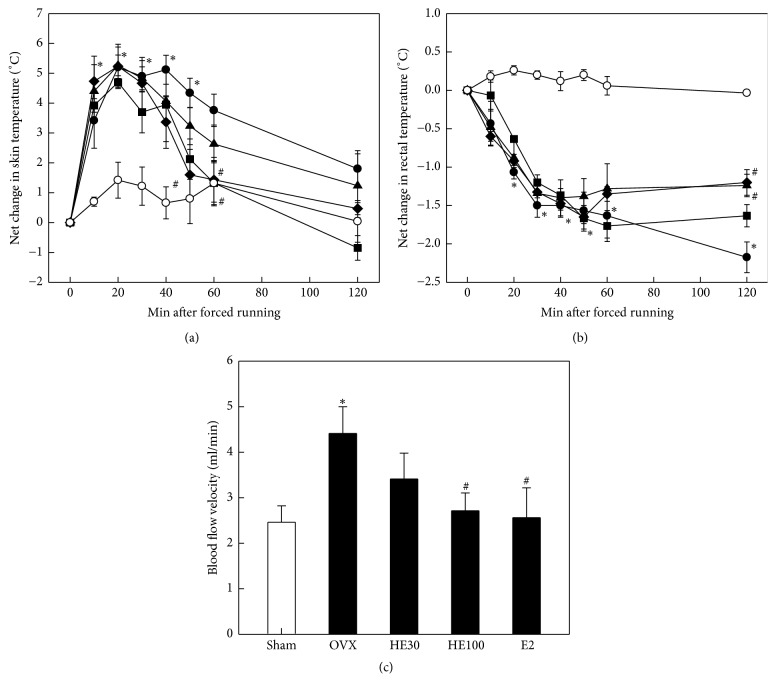
Effects of scheduled exercise training (Ex), hop extract (HE), and 17*β*-estradiol (E2) on the tail skin temperature (a), rectal temperature (b), and blood flow velocity (c) in ovariectomized (OVX) rats. ○: Sham-operation control, ●: OVX alone; ■: HE30 (30 mg/kg), ◆: HE100 (100 mg/kg), and ▲: E2 (100 *μ*g/kg). ^*∗*^Significantly different from Sham-operation control (*P* < 0.05). ^#^Significantly different from OVX alone (*P* < 0.05).

Tail skin temperature of OVX rats significantly increased following 15-min forced running up to 5.5°C (net increase) in 20–40 min, in comparison with 1.5°C in Sham-operated animals (*P* < 0.05) (Figure 4(a)). High temperature lasted for about 1 hour. Although HE pretreatment did not affect the exercise-induced early increase in skin temperature, it induced a rapid recovery in 50–60 min (*P* < 0.05). Also, E2 tended to advance the recovery phase of dermal fever. In contrast to the increase in skin temperature, rectal temperature significantly decreased in OVX animals after forced running and lasted longer than 2 hours (*P* < 0.05) (Figure 4(b)). A high dose (100 mg/kg) of HE and E2 significantly reversed the decreased rectal temperature in 2 hours.

As inferred from the increased skin temperature, tail blood flow velocity significantly increased after forced running in OVX rats (*P* < 0.05) (Figure 4(c)). Notably, the forced running-induced increase in blood flow was reduced by treatment of HE in a dose-dependent manner. E2 also reversed the increased blood flow velocity to the Sham-control level (*P* < 0.05).

## 4. Discussion

In the present study, HE treatment markedly attenuated postmenopausal symptoms; that is, oral administration of HE improved OVX-induced overweight, osteoporosis, and hot flash. Such effects were found to be due to the HE's activities regulating blood lipid profile and fat accumulation, blood estrogen and bone resorption factors, and dermal blood flow.

Postmenopausal estrogen deficiency causes various symptoms including hot flash, genital atrophy, osteoporosis, lipid metabolism disorders, depressive disorder, insomnia, and sexual dysfunction [2]. In OVX animals, we also observed the major symptoms such as increased blood lipids and overweight, skin blood flow and temperature, and bone resorption. According to serious side effects of HRT, a variety of phytoestrogens have been demonstrated to be effective in alleviating postmenopausal syndrome without considerable adverse-effects [12].

Hop extracts attracted investigators' attention, since they have unique prenylflavonoids such as 8-PN. 8-PN produced from IX by microflora in the human intestine is a potent ligand of estrogen receptor *α* [19, 20]. From the serial extraction procedures of supercritical CO_2_ conditions and 75% ethanol, we attained HE with 6% final yield containing 0.1–0.2% 8-PN.

Numerous studies have reported associations between postmenopausal status and elevated blood lipid levels, showing significant shift toward vascular risk factors [5, 6]. In the present study, OVX in rats markedly increased TG, TC, LDL, and LDL/HDL ratio, the major risk factors of obesity as well as cerebrovascular and cardiovascular diseases. Notably, such disturbances in lipid regulation were greatly recovered by treatment with HE. Expectedly, E2 drastically reduced the blood TC and LDL levels as well as LDL/HDL ratio, without affecting TG. However, E2 also decreased the level of HDL, a beneficial lipoprotein alleviating atherosclerosis and related vascular diseases. By comparison, scheduled exercise downregulated only TG level.

Down-regulation of the blood lipids decreased abdominal fat accumulation and body weight gain. Oral administration of HE remarkably attenuated the OVX-induced overweight in a dose-dependent manner by reducing perirenal, retroperitoneal, and mesenteric fats. Daily injection of E2 drastically reduced the body fats and body weights, which may be due to the depletion of blood lipids including HDL. Such a serious depletion of lipids, especially HDL, a lipoprotein beneficial for vascular diseases, by a long-term high dose of E2 my lead to adverse-effects of HRT [9, 10]. Notably, scheduled mild exercise also attenuated the fat accumulation and body weight gain, which may be achieved by the reduced blood TG level.

Menopause is well known to be associated with numerous physiological and biochemical changes affecting bone mineral metabolism [21]. In addition to Ca and P, serum ALP is a biomarker of bone formation denoting osteoblast activity. Thus, an elevated ALP activity is associated with bone turnover during menopause [22]. The depletion of 17*β*-estradiol in OVX rats significantly increased the blood ALP level, implying an active bone remodeling. Indeed, OVX lowered BMD and BMC of the femurs, decreasing their weights, the direct indicators of osteoporosis [23, 24]. The osteoporotic changes in the femoral bones of OVX rats were observed in microscopic examination: that is, depletion of estrogen caused severe loss of the trabecular bones and accumulation of multinuclear cells expressing TRAP, an osteoclast-specific enzyme, resulting in the deterioration of the trabecular architecture [25, 26].

Notably, daily treatment of HE recovered the blood estrogen level and thereby improved the osteoporotic changes, as evidenced by decreased blood ALP activity and bone TRAP immunoreactivity as well as restored trabecular bone mass with high BMD and BMC. Higher bone recovery was achieved with daily injection of E2, whereas the effects of scheduled exercise were relatively weak.

Menopausal vasomotor symptoms are caused by imbalanced thermoneutral zone of hypothalamus. Hot flash is the most prominent symptom induced by the change in hormone levels during menopause, which is experienced as a feeling of intense heat with sweating and rapid heartbeat. For that reason, peripheral skin temperature increases, but body temperature diminishes [8, 27]. Accordingly, treatment with 17*β*-estradiol, tibolone, or clonidine in estrogen-deficient rats reversed the raised tail skin temperature [28]. In the present study, the durations of change in tail and rectal temperatures of OVX rats following forced running were shortened by treatment with HE. In addition, the increased skin blood flow rate was downregulated by HE or E2 treatment, suggestive of their thermoregulatory functions via central thermoneutral and peripheral vasodilatating activities.

Taken together, we demonstrated beneficial effects of HE in the improvement of postmenopausal syndrome. The results indicate that HE remarkably attenuated overweight, osteoporosis, and hot flash in estrogen-deficient animals by regulating blood lipid profile and fat accumulation, blood estrogen and bone resorption factors, and dermal blood flow.

## Figures and Tables

**Table 1:** Effects of hop extract (HE) and 17*β*-estradiol (E2) on the blood lipids in ovariectomized rats.

Treatment	Sham	OVX	Ex	HE30	HE100	E2
TG (mg/dl)	65 ± 6.2	114 ± 2.9^*∗*^	62 ± 2.5^#^	94 ± 7.1	66 ± 6.6^#^	102 ± 19.9
TC (mg/dl)	112 ± 10.3	155 ± 15.6^*∗*^	124 ± 16.0	136 ± 2.4	123 ± 2.8^#^	90 ± 11.1^#^
LDL (mg/dl)	20 ± 4.6	35 ± 4.4^*∗*^	28 ± 6.8	23.7 ± 0.8^#^	26 ± 2.0^#^	9.0 ± 3.61^#^
HDL (mg/dl)	92 ± 6.9	117 ± 11.0	98 ± 11.7	107 ± 3.4	101 ± 2.0	77 ± 7.9^#^
LDL/HDL (%)	20.6 ± 3.6	30.1 ± 1.6	27.5 ± 3.4	22.1 ± 0.6^#^	25.6 ± 1.6	10.3 ± 3.4^#^

OVX: ovariectomy, Ex: scheduled exercise training, HE30: hop extract 30 mg/kg, HE100: hop extract 100 mg/kg, TG: triglycerides, TC: total cholesterols, LDL: low-density lipoproteins, and HDL: high-density lipoproteins. ^*∗*^Significantly different from Sham control (*P* < 0.05). ^#^Significantly different from OVX alone (*P* < 0.05).

**Table 2:** Effects of hop extract (HE) and 17*β*-estradiol (E2) on the factors affecting bone metabolism in ovariectomized rats.

Treatment	Sham	OVX	Ex	HE30	HE100	E2
17*β*-Estradiol (pg/ml)	2.9 ± 0.62	0.6 ± 0.44^*∗*^	1.7 ± 0.40	1.8 ± 0.32	2.4 ± 0.74^#^	2.6 ± 0.17^#^
ALP (U/ml)	49 ± 8.4	99 ± 8.5^*∗*^	63 ± 12.9	57 ± 13.9^#^	52 ± 1.8^#^	73 ± 5.4
Ca (mg/dl)	10.2 ± 0.34	10.0 ± 0.40	9.5 ± 0.17	9.9 ± 0.11	9.8 ± 0.04	9.8 ± 0.05
P (mg/dl)	5.7 ± 0.71	3.9 ± 0.05^*∗*^	3.9 ± 0.55	4.4 ± 0.37	4.6 ± 0.33	4.3 ± 0.54

OVX: ovariectomy, Ex: scheduled exercise training, HE30: hop extract 30 mg/kg, HE100: hop extract 100 mg/kg, and ALP: alkaline phosphatase. ^*∗*^Significantly different from Sham control (*P* < 0.05). ^#^Significantly different from OVX alone (*P* < 0.05).
