# Corrigendum to “A Hop Extract Lifenol® Improves Postmenopausal Overweight, Osteoporosis, and Hot Flash in Ovariectomized Rats”

**DOI:** 10.1155/2018/4503614

**Published:** 2018-06-24

**Authors:** Young-Hwan Ban, Jung-Min Yon, Yeseul Cha, Jieun Choi, Eun Suk An, Haiyu Guo, Da Woom Seo, Tae-Su Kim, Sung-Pyo Lee, Jong-Choon Kim, Ehn-Kyoung Choi, Yun-Bae Kim

**Affiliations:** ^1^College of Veterinary Medicine and Veterinary Medical Center, Chungbuk National University, Cheongju, Republic of Korea; ^2^Anydoctor Healthcare Co., Ltd., Cheonan, Republic of Korea; ^3^College of Veterinary Medicine, Chonnam National University, Gwangju, Republic of Korea

In the article titled “A Hop Extract Lifenol® Improves Postmenopausal Overweight, Osteoporosis, and Hot Flash in Ovariectomized Rats” [1], there was a missing grant, which should be added to the Acknowledgments section as follows.

This work was supported by the intramural research grant of Chungbuk National University in 2015.
